# Mild cognitive impairment in patients with Parkinson’s disease: An updated mini-review and future outlook

**DOI:** 10.3389/fnagi.2022.943438

**Published:** 2022-09-06

**Authors:** Rwei-Ling Yu, Ruey-Meei Wu

**Affiliations:** ^1^College of Medicine, Institute of Behavioral Medicine, National Cheng Kung University, Tainan, Taiwan; ^2^Department of Neurology, College of Medicine, National Taiwan University Hospital, National Taiwan University, Taipei, Taiwan

**Keywords:** Parkinson’s disease, dementia, mild cognitive impairment, heterogeneity, cognitive function

## Abstract

Mild cognitive impairment (MCI) is one of the common non-motor symptoms in patients with Parkinson’s disease (PD). MCI is the transition stage between normal aging and full-blown dementia and is also a powerful predictor of dementia. Although the concept of MCI has been used to describe some of the PD symptoms for many years, there is a lack of consistent diagnostic criteria. Moreover, because of the diverse patterns of the cognitive functions, each cognitive impairment will have a different progression. In this review, we overviewed the diagnostic criteria for PD-MCI, primarily focused on the heterogeneity of PD-MCI patients’ cognitive function, including various types of cognitive functions and their progression rates. A review of this topic is expected to be beneficial for clinical diagnosis, early intervention, and treatment. In addition, we also discussed the unmet needs and future vision in this field.

## Introduction

Parkinson’s disease (PD) is one of the common neurodegenerative diseases. Some critical clinical features include motor symptoms, such as tremors, bradykinesia, and rigidity (Obeso et al., 2017). Evidence showed that there were 6.1 million PD patients worldwide in 2016, and the prevalence continues to rise each year (Dorsey et al., 2018). PD is more common in males, and there is a slightly higher incidence and prevalence rate of PD in the West compared to the East (Abbas et al., 2018). The clinical diagnostic criteria of PD have been updated continuously (Gibbs and Lees, 1988; Gelb, 1999; Postuma et al., 2015; Marsili et al., 2018). New aspects are introduced in the updated diagnostic guidelines, such as the use of non-motor symptoms (NMSs) and the application of the prodromal concept, which is essential for the early detection and treatment of the disease (Marsili et al., 2018).

In addition to motor symptoms, NMSs are common in patients with PD across cultures and countries (Yu et al., 2017). The NMSs may precede the development of motor features and have a more significant impact on a patient’s quality of life (Liu et al., 2015; Pfeiffer, 2016). It may also serve as a predictor of mortality (Bugalho et al., 2019). PD’s common NMSs include dementia, neuropsychiatric symptoms, autonomic failure, and sensory impairments (Gupta and Shukla, 2021). Among the NMSs, dementia has the most detrimental effect on patients’ quality of life (Fan et al., 2020), caregivers’ burden (Pantula and Vijay Harbishettar, 2012), and increases the need for hospitalization and mortality (Bugalho et al., 2019).

The clinical diagnostic criteria for PD patients with Dementia (PDD) were first published by the movement disorder society (MDS) task force (Emre et al., 2007), and then another practical guideline for diagnosing PDD using two-level criteria was proposed (Dubois et al., 2007). A review by Aarsland and Kurz (2010) showed that the point prevalence of dementia in the PD population is about 30%, and the incidence rate of dementia was 24.3/1,000 per year (95% confidence interval is 7.7–58.5) in a hospital-based PD cohort (Nicoletti et al., 2019). PD patients are 5∼6 times more likely to develop dementia than healthy aging populations (Hobson and Meara, 2004). Moreover, the longitudinal studies showed that 15–20% of PD patients develop dementia after 5 years (Williams-Gray et al., 2009) and 46% after 10 years (Williams-Gray et al., 2013). Although dementia does not necessarily occur in PD, the 12-year (Buter et al., 2008) and 20-year follow-up studies (Hely et al., 2008) revealed that about eighty percent of PD patients are eventually diagnosed with dementia. Mild cognitive impairment (MCI) is believed to be one of the best indicators for early detection of PDD (Galtier et al., 2016; Hoogland et al., 2017; Nicoletti et al., 2019). Therefore, the number of studies investigating PD patients with MCI (PD-MCI) has been significantly growing for the past 20 years.

## Mild cognitive impairment in patients with Parkinson’s disease

### The evolution of the concept and the diagnostic criteria of Parkinson’s disease patients with mild cognitive impairment

The concept of MCI comes from Alzheimer’s disease research, and it is believed to be the transition stage between normal aging and dementia (Petersen et al., 1999; Petersen, 2004). The concept of MCI is also incorporated into the last version (fifth edition) of the Diagnostic and Statistical Manual (DSM) system of the mental disorder (Regier et al., 2013), in which neurocognitive disorders (NCD) are divided into major NCD and mild NCD according to whether the patient’s independent living function is affected. Suppose the patient’s social or occupational function is intact, but there is a deficit in cognitive function. In that case, the patient will be diagnosed with mild NCD, which is a synonym for MCI.

After the concept of MCI was introduced into PD research, many scholars and research teams began to explore the neurocognitive function profile of PD patients and its progression with the course of the disease through different definitions or diagnostic criteria of MCI. Most studies (Janvin et al., 2006; Monastero et al., 2012; Yu et al., 2012b) followed the diagnostic criteria from the original MCI criteria from Petersen (2004). In 2011, the Movement Disorder Society commissioned a Task Force to systematically review the literature and determine the PD-MCI patient’s clinical characteristics (Litvan et al., 2011). After Litvan et al. (2012) proposed the standardized diagnostic criteria for PD-MCI, in the next 10 years, most of the PD-MCI related studies deployed this diagnostic criterion, and validation was conducted (Geurtsen et al., 2014). There is a two-level scheme (i.e., level I and II) in this standardized diagnostic criteria for PD-MCI (Litvan et al., 2012). In level I, the abbreviated testing tools are used to judge the patient’s cognitive performance. Level II uses various cognitive domains (e.g., memory, executive, visuospatial function, etc.) to determine the patient’s cognitive function; each cognitive domain contains at least two cognitive tests. Level II can be used to classify patients with PD-MCI to explore the heterogeneity of PD-MCI further. The classification methods include using the number of cognitive domain deficits as a classification, such as single-domain or multiple-domain subtypes. Another classification method is to use cognitive impairment content as a classification method, such as amnestic or non-amnestic subtypes. The Level I is often recommended for clinical practice, while Level II using comprehensive neuropsychological tests is recommended for research use. Recently, Goldman et al. (2018a) revisited the concept of MCI and the international Parkinson and MDS PD-MCI diagnostic criteria. They pointed out that using different diagnostic criteria (e.g., Level I or Level II) will lead to the different prevalence of PD-MCI. Which cognitive test is used and how the defect is defined (e.g., using 1 or 2 SD) can affect the diagnostic classification of PD-MCI. Most studies indicate that the use of −2SD will have the best sensitivity, and the proportion of PD-MCI with multiple domains was the most common. Delineation of PD-MCI cognitive subtypes is crucial for predicting cognitive decline and responding to associated pathological changes. Cognitive tests and other functional assessments play an essential role in the diagnosis, and Goldman et al. (2015) suggest related clinical and psychometric properties of scales should be considered in the diagnostic criteria.

### Prevalence, progression, and subtype of Parkinson’s disease patients with mild cognitive impairment

Litvan et al. (2011) conducted a critical systematic review and disclosed that about 26.7% (18.9–38.2%) of non-demented PD patients have MCI. Yarnall et al. (2014) applied different cut-off criteria of cognitive tests and found various PD-MCI prevalence; their results showed that under 1, 1.5, and 2 standard deviations, the prevalence of PD-MCI was 65.8, 42.5, and 22.4%, respectively (Yarnall et al., 2014). Recently, Baiano et al. (2020) conducted a meta-analysis study to elucidate the prevalence of MCI in PD. The authors recruited forty-one studies (7,053 PD patients) and found that the prevalence of MCI in PD was around 40% (95% confidence interval is 36–44). Moreover, this meta-analysis study revealed that the multiple-domain subtype was the most common phenotype of PD-MCI (about 31%) (Baiano et al., 2020). Nicoletti et al. (2019) enrolled a hospital-based cohort and showed the incidence rate of MCI among PD patients was 184.0/1,000 per year (95% confidence interval is 124.7–262.3).

Janvin et al. (2006) first used a small sample (72 non-demented PD patients) to explore this topic and found that sixty-two percent of PD-MCI patients developed PDD over 4 years (Janvin et al., 2006). Although it is difficult to compare all these studies due to the various research designs or methodologies, an updated review and meta-analysis article was done by Saredakis et al. (2019) to elucidate the conversion rate of PD-MCI to PDD. They included 39 articles (4,011 PD patients) in this study. They found that about 25% of PD patients converted to PD-MCI and 2% to dementia among the patients with intact cognitive function. Besides, 20% of PD-MCI converted to dementia, while 28% reverted to normal cognitive function. Here we summarized the primary longitudinal studies that elucidated the trajectory of cognitive function in patients with PD in Table 1 (Broeders et al., 2013; Pedersen et al., 2013, 2017; Hobson and Meara, 2015; Pigott et al., 2015; Santangelo et al., 2015; Galtier et al., 2016).

**TABLE 1:** The longitudinal studies explore the trajectory of cognitive function in patients with PD.

References	Country	Center	Dropout rate	Follow-up year	PDCN→PD-MCI	PD-MCI→PDD
Broeders et al. (2013)	Holland	Single	21.1%	3	36.5	17.6
			40.7%	5	−	−
Pedersen et al. (2013)	Norway	Multi	8.2%	3	−	27%
Pigott et al. (2015)	United States	Single	19.1%	4	36.1%	78.7%
Santangelo et al. (2015)	Italy	Single	18.4%	2	29.2%	0%
			27.6%	4	33.3%	15.3%
Hobson and Meara (2015)	United Kingdom	Single	37.3%	4	40.5%	85.7%
			45.2%	6	62.5%	53%
Galtier et al. (2016)	Spain	Single	9.3%	7	−	42.3%
Pedersen et al. (2017)	Norway	Multi	1.69%	1	10.1%	0%
			8.43%	3	21.5%	34.5%
			15.73%	5	14.3%	38.5%

PD, Parkinson’s disease; PDCN, PD patients without dementia; PD-MCI, PD patients with mild cognitive impairment; PDD, PD patients with dementia.

The heterogeneity of the PD-MCI patients has long been noted (Kehagia et al., 2010). The original MCI concept proposed the amnestic and non-amnestic or the single or multiple domains impaired (Petersen, 2004). The MDS PD-MCI diagnostic criteria proposed a two-level diagnostic method. Level I is suggested for clinical practice, and level II is for research. The most commonly used tests for level I are the Mini-mental state examination or Montreal Cognitive Assessment. The conversion equation between the two tests was recently proposed (Yu et al., 2020). Level II is a more comprehensive evaluation that examines various cognitive domains and can be applied to classify different subtypes of PD-MCI (Litvan et al., 2012). Although various studies investigated the neuropsychological profile in PD-MCI patients (Sollinger et al., 2010; Goldman et al., 2012; Yu et al., 2012b; Galtier et al., 2016; Kalbe et al., 2016; Lawrence et al., 2016; Monastero et al., 2018); the results were inconclusive. Some studies showed that single domain PD-MCI is the most frequent subtype (Sollinger et al., 2010), especially the non-amnestic type, and the executive and visuospatial functions were the most vulnerable (Goldman et al., 2012; Yu et al., 2012b; Kalbe et al., 2016). On the other hand, other studies demonstrated that multiple domain impairment was the most common subtype (Galtier et al., 2016; Lawrence et al., 2016; Monastero et al., 2018). Recently, a updated meta-analysis study conducted by Baiano revealed that the multiple domain subtype was the most common phenotype of PD-MCI (about 31%) (Baiano et al., 2020). Janvin et al. (2006) found that non-amnestic PD-MCI was associated with the later development of dementia, whereas amnestic PD-MCI was not (Janvin et al., 2006). However, this finding was not supported by other studies. Chung et al. (2019) found that amnestic PD-MCI patients exhibited a more rapid cognitive deterioration in executive function than non-amnestic PD-MCI patients. Moreover, the amnestic PD-MCI group had a higher risk of converting to dementia than the non-amnestic PD-MCI group (Chung et al., 2019). In addition, Vasconcellos et al. (2019) showed that the amnestic PD-MCI patients have the worst quality of daily life.

## The unmet need and future outlook

Different PD-MCI diagnostic criteria will generate different prevalence rates. The estimated prevalence of PD-MCI using MDS criteria is approximately 40% (Baiano et al., 2020). The prevalence may be overestimated (Monastero et al., 2012; Yu et al., 2012b) or underestimated (Muslimović et al., 2005; Aarsland et al., 2010) depending on the diagnostic criteria used. In recent years, most studies have used the diagnostic criteria which were proposed by the MDS Taskforce (Litvan et al., 2012); however, these criteria are still under modification. The PD-MCI diagnostic criteria can be revised and refined by considering other biomarkers and possible factors (e.g., gender or effective measurement) to increase the accuracy. For example, the Catechol-O-methyltransferase genotype was found to be related to executive-attention function (Foltynie et al., 2004; Williams-Gray et al., 2009; Fang et al., 2019), and the apolipoprotein E genotype is related to cognitive decline (Tropea et al., 2018), especially the posterior cortical dysfunction (Williams-Gray et al., 2009), in the PD population. Martinez-Horta and Kulisevsky first proposed two subtypes (i.e., frontostriatal and posterior-cortical cognitive defects) in patients with PD. They suggested that one subtype is frontostriatal cognitive dysfunction and the frontostriatal dopaminergic deficits leading to the dysexecutive syndrome and that this deficit may be a benign and non-progressive subtype. Furthermore, the other subtype is tissue damage due to the spread of Lewy bodies, while damage to specific functions (e.g., visuospatial and language functions) is dependent on posterior cortical areas and represents a malignant and progressive subtype (Martínez-Horta and Kulisevsky, 2011). That is, the posterior cortical dysfunction is related to the progression of dementia; however, this is not the case with the frontal-related dysfunction. Later, Kehagia et al. (2013) proposed the “dual syndrome hypothesis” to describe the heterogeneity of neurocognitive function in patients with PD. In addition, certain race-specific genes (e.g., Aldehyde Dehydrogenase) (Yu et al., 2016, 2021) and other genes (e.g., β-glucocerebrosidase) (Szwedo et al., 2022) are associated with cognitive function should also be considered and further investigate.

Moreover, brain imaging is another potential biomarker. Evidence showed that PD-MCI patients’ brain atrophy mainly occurs in the frontal, temporal and parietal regions and the basal forebrain (Delgado-Alvarado et al., 2016). Through whole-brain analysis (e.g., Voxel-based meta-analysis or coordinate-based meta-analysis), the cross-sectional studies revealed that PD-MCI patients have more atrophy in the left brain areas, including superior frontal gyrus, superior temporal lobe, and insula (Xu et al., 2016), left anterior insula extending to the inferior frontal gyrus, and orbital region (Zheng et al., 2019), angular gyrus, and right supramarginal gyrus, bilateral dorsolateral prefrontal cortex, and midcingulate cortex (Mihaescu et al., 2019). The longitudinal research showed that baseline volume of global white matter, global hippocampus, hippocampal sub-regions, thalamus, and accumbens nucleus are predictors of the PD patients with normal cognition (PDNC) conversion to PD-MCI (Atluri et al., 2013; Kandiah et al., 2014; Wen et al., 2015). While the baseline global gray matter volume cannot significantly predict the conversion rate to PD-MCI, one side to bilateral loss of gray matter volume is a predictor of progression from PD-MCI to PDD (Xu et al., 2016). The functional MRI studies showed that the functional connectivity between the medial prefrontal and posterior cingulate cortex within the default mode network at baseline predicts PD patients’ conversion to PD-MCI (Zarifkar et al., 2021). PD-MCI patients have reduced connectivity in specific brain regions that are part of the default mode network (Wolters et al., 2019), and the functional connectivity changes involving the parieto-temporal regions may predict the evolution of dementia in PD-MCI patients (Dubbelink et al., 2014). In addition, cerebral blood flow abnormality is a detection marker for PD-MCI patients. Nobili et al. (2009) used single-photon emission computed tomography to evaluate the perfusion in patients with PD and found that PD-MCI patients have the hypo-perfusion pattern in the posterior brain area (e.g., bilateral posterior parietal lobe and right occipital lobe) compared with healthy individuals. The parietal cerebral blood flow was found to be a potential early biomarker for PD-MCI (Pelizzari et al., 2020). Recently, Arslan et al. (2020) found a “posterior hypo-perfusion” pattern *via* arterial spin labeling imaging (ASL-MRI), and this pattern can be differentiated PD-MCI from healthy individuals with an accuracy of 92.6%. Moreover, PD patients with microtubule-associated protein tau (MAPT) H1/H1 haplotype had decreased perfusion than the ones with H1/H2 haplotype in the posterior brain regions. They suggested that “posterior hypo-perfusion” in ASL-MRI could potentially be a biomarker for detecting cognitive dysfunction in the PD population (Arslan et al., 2020). Azamat et al. (2021) also found that the abnormities of cerebral blood flow are very different between PDD and non-demented PD patients (i.e., PDNC and PD-MCI). They found PDD patients especially have hypoperfusion in dopaminergically-mediated fronto-parietal and non-dopaminergically-mediated visual networks (Azamat et al., 2021). Those imaging studies suggest a dual characteristics of cognitive impairment (i.e., the dopaminergic fronto-striatal pathway and the parieto-temporal pathway (Kehagia et al., 2010, 2013). Future studies could target the combination of multiple biomarkers (e.g., imaging and genetics) to detect the occurrence of PD-MCI and predict the subsequent development of PD-MCI.

The impact of gender on a patient’s cognitive function should not be underestimated. Male sex is a significant predictor of early cognitive decline, and females have slower progression to cognitive impairment (Cholerton et al., 2018). Evidence showed that gender was a significant determinant of specific cognitive domains, with a differential pattern of decline in male and female PD patients. Moreover, how to efficiently measure the functional deficit is another crucial issue. One is that functional deficits other than cognitive function have yet to be developed, and the other is the method in which function is assessed. The former refers to the fact that, in addition to cognitive function, PD patients have many functional impairments in life, such as instrumental activities of daily living (Pirogovsky et al., 2014) or interpersonal/social function (Perepezko et al., 2019; Su et al., 2020; Chuang et al., 2021). A few studies have explored this topic; however, research in these areas still requires more relevant studies and the accumulation of empirical data.

Regarding the way to measurement, common assessment methods include self-report, performance-based measurement, or informant-based measurement. Self-report and family reports rely on the reporter’s observation. Evidence showed that not all PD patients could be precisely aware of their dysfunction due to the brain basis of the disease (Yu et al., 2010). The informant-based measurement may have more uncontrollable factors, including family members not living together (patients live alone) or family members’ poor observation. In addition, the biggest challenge for performance-based measurement is the ecological validity of the test (Lea et al., 2021). Ecological validity refers to the degree to which the test content can reflect the actual living environment of the patient. Good ecological validity can improve the usability of assessment for diagnosis or treatment. The ecological validity of performance-based measurement is a key that needs to be studied in depth.

Past diagnostic criteria have focused on the contents of cognitive tests (Hoogland et al., 2018) or the optimal cut-off score of the cognitive tests (Goldman et al., 2013, 2015), but how would cognitive impairments reflect difficulties in PD patients’ life? This question needs to be explored urgently. The ecological validity of cognitive tests needs to be noted, and it is also essential to develop tools that can assess the distress experienced by patients in their life. For example, the social function deficits would escalate a person’s risk of dementia (Fankhauser et al., 2015) and expedite the dementia process (Bennett et al., 2006). This issue has gradually been noticed in the PD group (Bettencourt and Sheldon, 2001; Perepezko et al., 2019). The tools for measuring PD patients’ social functioning were developed (Su et al., 2020). Developing such measurement and in-depth knowledge of related fields will help tailor rehabilitation programs for PD patients. It is particularly worth noting that during the COVID-19 pandemic, PD patients may be at particular risk for developing new cognitive symptoms or worsening existing cognitive symptoms, even if the patients are not infected with COVID-19 (Brown et al., 2020). Brown et al. (2020) revealed that PD patients may experience worsening or new symptoms of cognitive function after being canceled or postponed exercise or social activities or being asked to self-isolate/quarantine during the COVID-19 pandemic. In the past 2∼3 years, human-to-human contact has been limited. Under such conditions, the deterioration rate of the social function of PD patients may increase. Moreover, PD patients’ social function performance in the post-epidemic period is also worth further study.

Since the brain pathology of each PD-MCI subtype may be different, the clinical characteristics of the PD-MCI subtype may be different too. PD patients have specific motor characteristics, and the correlation between these motor characteristics and PD-MCI subtypes needs to be explored. For example, a recent study demonstrated that multiple-domain PD-MCI and amnestic PD-MCI are related to gait disturbance, especially in the dual-task (Amboni et al., 2022). Our study also revealed that the PD-specific motor characteristic (e.g., hypomimia) might influence social cognition (i.e., facial emotion recognition) (Chuang et al., 2021). Based on the embodied simulation theory, the mechanism for understanding the thoughts and emotions of others is simulation through the mirror mechanism (Gallese et al., 2004). People can trigger sensorimotor neurons by simulating other people’s facial expressions, followed by a series of responses to complete emotion recognition (e.g., triggering proprioceptive feedback, generating corresponding emotional states, recognizing emotions). Our findings revealed that PD patients with hypomimia had worse recognition of disgust than healthy aging, and hypomimia’s severity was predictive of the recognition of disgust.

Different from other cognitive functions (e.g., memory, executive function, etc.), social cognition (e.g., facial emotion recognition, reading the mind in the eye, theory of mind, etc.) is an emerging research field (Regier et al., 2013) and has drawn more attention in neurodegenerative disease (Elamin et al., 2012), especially in the PD population (Lewis and Ricciardi, 2021). Social cognition refers to people’s understanding and prediction of themselves and others by processing and using the information in social interaction and then forming the interactive behavior between people and themselves, including the cognitive and affective components. These components differentially served in distinct neural circuits (Shamay-Tsoory and Aharon-Peretz, 2007). Activity in the dorsolateral prefrontal cortex, posterior cingulate cortex, and the temporoparietal junction is responsible for the cognitive part of social cognition, while the affective domain relies on ventromedial, orbitofrontal cortices, and the mesolimbic circuit (Schurz et al., 2014). The Diagnostic and Statistical Manual of Mental Disorders first juxtaposes social cognition with other cognitive functions (Regier et al., 2013). One main research directions in this field are the theory of mind (ToM) (Yu et al., 2012a,^2018^; Yu and Wu, 2013a,b; Argaud et al., 2018; Adenzato et al., 2019; Foley et al., 2019; Romosan et al., 2019; Coundouris et al., 2020). ToM refers to individuals’ ability to know what others think (cognitive ToM) and how they feel (affective ToM). Evidence showed that young-onset PD patients had preserved ToM (Yu et al., 2018); however, the idiopathic PD patients have impaired cognitive ToM, and the affective ToM will be affected in the advanced stages of the disease (Poletti et al., 2011; Bora et al., 2015).

Moreover, the deficits in affective ToM may make more significant in female than male PD patients (Yu et al., 2018). Recently, Coundouris et al. (2020) conducted a meta-analysis of 38 studies and revealed that cognitive or affective ToM only evident for performance-based tests. Moreover, Affect ToM in PD patients was less affected than cognitive ToM, but since there has been less research on this topic, the authors believe this finding still needs to be validated in future studies. Only a few studies were conducted to investigate the PD-MCI patient’ theory of mind to the best of our knowledge. Adenzato et al. (2019) compared the ToM performance in twenty patients with PD-MCI and healthy controls. They found that PD-MCI patients’ ToM performance was worse than that in the healthy controls. They also found that transcranial direct over the medial frontal cortex enhances ToM in PD-MCI patients; however, no effect on accuracy was observed. The limitation of the small sample size and methodology (e.g., classified PD-MCI on a global scale) may limit the application of results (Adenzato et al., 2019). The relationship between cognitive function and social cognition is still unclear. Some studies suggested that cognitive function and social cognition are two separate concepts that do not affect each other (Roca et al., 2010); however, other evidence showed that impairment in ToM might be explained by cognitive function (e.g., executive function and attention and visuospatial function) (Yu et al., 2012a; Bora et al., 2015; Foley et al., 2019; Romosan et al., 2019). If social cognition is inseparable from cognitive function, then it is conceivable that PD-MCI patients may have impaired social cognition. In the process of social cognition, cognitive function plays a key role is important. If expressing appropriate responses in social situations may require assistance with cognitive functions (e.g., inhibition or monitoring abilities), training these cognitive functions will help patients maintain good social cognition. Considering the heterogeneity of PD-MCI, it is also considered that preserved social cognition helps patients adhere to physician orders, maintain relationships with caregivers, and maintain quality of life during the disease course. Further research is needed to explore the relationship between cognitive function and social cognition.

**FIGURE 1:**
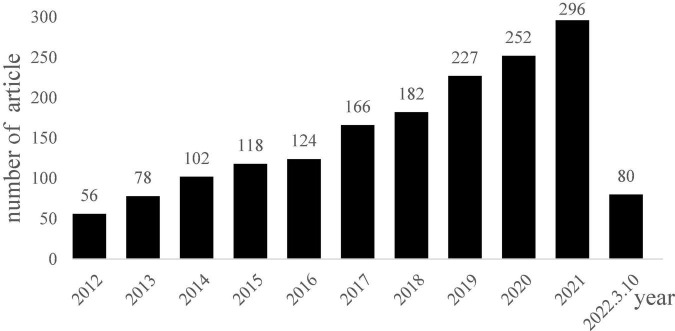
Yearly articles related to cognitive training for Parkinson’s disease on PubMed.

Regarding the treatment of cognitive impairment in patients with PD, most of the treatment trials for dementia in patients with PD have focused on the development of drugs which was developed for the treatment of cognitive symptoms in Alzheimer’s disease, including cholinesterase inhibitors and the N-methyl-D-aspartate receptor antagonist, memantine (Goldman and Weintraub, 2015). The cholinesterase inhibitors were evidence-based for the symptomatic treatment for PDD; however, less evidence is available for memantine (Seppi et al., 2011; Dubois et al., 2012). A recent meta-analysis was conducted to examine the efficacy of cholinesterase inhibitors and memantine for PDD and Lewy body dementia. A total of fifteen trials were recruited, and the results showed that cholinesterase inhibitors had effects on some cognitive functions (e.g., attention, processing speed, executive function, memory, and language); however, there was no significant effect on improving visuospatial perception. Memantine also significantly affected attention, processing speed, and executive functions (Meng et al., 2019). However, the authors suggested further clinical trials are required to verify their conclusions due to the few studies included in this study. Given the side effect of the medication and the lack of pharmacological treatments for MCI in PD, non-pharmacological treatments have attracted great interest in recent years (Goldman et al., 2018b). We searched and screened the literature from 2012 to 2022 in the PubMed database through the keywords “Parkinson’s disease “&” Cogni* training,” and excluded review articles, meta-analysis articles, articles that did not investigate cognitive training, and did not evaluate the changes of cognitive function. We found 1,679 articles in total, and the number of articles is increasing yearly (Figure 1).

The cognitive training methods can be divided into traditional cognitive training (or paper-pencil tasks training) and computerized cognitive training (Pupíková and Rektorová, 2020; Svaerke et al., 2020). Researchers began using computers from 2013 to 2014 to aid cognitive training. However, few studies were conducted on patients with PD-MCI (Costa et al., 2014; Angelucci et al., 2015). Three randomized controlled trials were conducted to evaluate the effect of cognitive training in the PD-MCI population. The research team of Costa et al. (2014) and Angelucci et al. (2015) recruited 15 and 17 PD-MCI patients, respectively, through the level II of the PD-MCI diagnostic criteria for paper-pencil cognitive training. The executive function (e.g., mental shifting) is the common focus of the two studies, while the study by Costa et al. (2014) additionally trained attention and working memory ability. After 4 weeks (12 sessions) of training, the patients’ performance in the zoo map test, the trial-making test, and prospective memory were improved. In 2014, Cerasa et al. (2014) recruited 15 PD-MCI patients according to the level I of PD-MCI criteria; they used a computerized training program to train patients’ attention ability. They found that after 6 weeks (12 sessions) of training, the patients’ attention (e.g., digit span and the symbol digit modalities test) were improved. Although these studies revealed that cognitive training might help PD-MCI patients to improve their cognitive function; however, the evidence level of these articles was classified as “possibly effective or ineffective” (Pupíková and Rektorová, 2020). The limited number of randomized controlled trials for PD-MCI patients’ cognitive training makes it difficult to draw further conclusions. More studies on cognitive training were warranted, especially developing the cognitive training for other vulnerable cognitive domains (e.g., visuospatial function). Tailed training program to use a preserved cognitive function to assist impaired one. For example, we found impaired gist memory in advanced-stage but not early stage PD patients. The techniques used to take advantage of the preserved gist memory in early stage patients with PD and the preserved item-specific memory in patients with PD of all stages could be helpful for the memory training program (Yu et al., 2015).

Moreover, develop cognitive training programs through other equipment as a medium. For example, some researchers applied virtual reality technology (Pelosin et al., 2021) to enhance the sense of reality and used mobile applications (Yu et al., 2022) to improve the accessibility of cognitive training. Last, the prospective and longitudinal designed studies were also urgent to evaluate the long-term effects of cognitive training.

**FIGURE 2:**
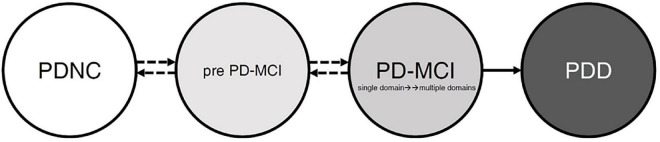
The possible patterns and evolution of cognitive function in patients with Parkinson’s disease. Before full-blown dementia, the PD patients’ cognitive patterns may be in the “PDNC,” “pre PD-MCI,” or “PD-MCI.” The pathway from PD-MCI to PDD is relatively stable; however, the three cognitive states may transition before entering into PDD. PDNC, PD patients with normal cognition; pre PD-MCI, PD patients do not achieve PD-MCI diagnosis but have cognitive impairment; PD-MCI, PD patients achieve PD-MCI diagnosis; PDD, PD patients with dementia.

Last but not least, the cognitive state transitions during PD are noteworthy. We proposed possible transition states for cognitive functions (see Figure 2). The state includes the “PD patients with normal cognition,” “pre PD-MCI,” “PD-MCI,” and “PDD.” Many studies have confirmed the pathway for the conversion of PD-MCI to PDD (Galtier et al., 2016; Hoogland et al., 2017; Nicoletti et al., 2019), and once a patient is diagnosed with PDD, it means that the course of the disease will not be reversed back to PD-MCI. However, before developing PDD, we assume that there is a possibility of mutual conversion between these stages. For example, “PD-MCI” state converts back to “pre PD-MCI” or “pre PD-MCI” reverse to “PDNC.” According to the diagnostic criteria of PD-MCI in MDS (Litvan et al., 2012), there may also be a “pre PD-MCI” group of PD patients. The “pre PD-MCI” is a less mentioned group, this group of patients has not yet met the diagnostic criteria of PD-MCI, but they have cognitive deficits (with one test score falling within the deficit range) (Goldman et al., 2018a). To our knowledge, no study was conducted to elucidate this group’s characteristics and conversion or reversion rate. Future research will be encouraged to explore the characteristics of different states and the transition of each state as a basis for brain pathology research, and the findings can also provide a reference for rehabilitation planning.

## Author contributions

R-MW and R-LY formed the concept and structure of the manuscript together. R-LY wrote the first draft of the manuscript. R-MW supervised, commented, revised, and critiqued this manuscript. Both authors approved the final version of the manuscript and agreed to be responsible for all aspects of the work.
